# Reliability of predictive models to support early decision making in the emergency department for patients with confirmed diagnosis of COVID-19: the Pescara Covid Hospital score

**DOI:** 10.1186/s12913-022-08421-4

**Published:** 2022-08-19

**Authors:** Ennio Polilli, Antonella Frattari, Jessica Elisabetta Esposito, Milena D’Amato, Giorgia Rapacchiale, Angela D’Intino, Alberto Albani, Giancarlo Di Iorio, Fabrizio Carinci, Giustino Parruti

**Affiliations:** 1 Clinical Pathology Unit, Pescara General Hospital, Pescara, Italy; 2 Unit of Intensive Care, Pescara General Hospital, Pescara, Italy; 3 Department of Pharmacy, Pescara General Hospital, Pescara, Italy; 4 Emergency Department, Pescara General Hospital, Pescara, Italy; 5 Department of Statistical Sciences, Università di Bologna, Bologna, Italy; 6 Infectious Diseases Unit, Pescara General Hospital, Pescara, Italy

**Keywords:** COVID-19, Prognosis, Prediction score, Decision making, Intensive care, Hospitalisation, Survival

## Abstract

**Background:**

The hospital management of patients diagnosed with COVID-19 can be hampered by heterogeneous characteristics at entry into the emergency department. We aimed to identify demographic, clinical and laboratory parameters associated with higher risks of hospitalisation, oxygen support, admission to intensive care and death, to build a risk score for clinical decision making at presentation to the emergency department.

**Methods:**

We carried out a retrospective study using linked administrative data and laboratory parameters available in the initial phase of the pandemic at the emergency department of the regional reference hospital of Pescara, Abruzzo, Italy, March–June 2020. Logistic regression and Cox modelling were used to identify independent predictors for risk stratification. Validation was carried out collecting data from an extended timeframe covering other variants of concern, including Alpha (December 2020–January 2021) and Delta/Omicron (January–March 2022).

**Results:**

Several clinical and laboratory parameters were significantly associated to the outcomes of interest, independently from age and gender. The strongest predictors were: for hospitalisation, monocyte distribution width ≥ 22 (4.09; 2.21–7.72) and diabetes (OR = 3.04; 1.09–9.84); for oxygen support: saturation < 95% (OR = 11.01; 3.75–41.14), lactate dehydrogenase≥237 U/L (OR = 5.93; 2.40–15.39) and lymphocytes< 1.2 × 10^3^/μL (OR = 4.49; 1.84–11.53); for intensive care, end stage renal disease (OR = 59.42; 2.43–2230.60), lactate dehydrogenase≥334 U/L (OR = 5.59; 2.46–13.84), D-dimer≥2.37 mg/L (OR = 5.18; 1.14–26.36), monocyte distribution width ≥ 25 (OR = 3.32; 1.39–8.50); for death, procalcitonin≥0.2 ng/mL (HR = 2.86; 1.95–4.19) and saturation < 96% (HR = 2.74; 1.76–4.28). Risk scores derived from predictive models using optimal thresholds achieved values of the area under the curve between 81 and 91%. Validation of the scoring algorithm for the evolving virus achieved accuracy between 65 and 84%.

**Conclusions:**

A set of parameters that are normally available at emergency departments of any hospital can be used to stratify patients with COVID-19 at risk of severe conditions. The method shall be calibrated to support timely clinical decision during the first hours of admission with different variants of concern.

**Supplementary Information:**

The online version contains supplementary material available at 10.1186/s12913-022-08421-4.

## Background

Between December 2019 and April 2021, the coronavirus SARS-CoV2 affected at least 130 Million people diagnosed with COVID-19 in 223 different countries, of which almost 2.9 Million died [1]. The range of complications associated with the disease generated enormous pressure on hospitalisation and intensive care [2–4].

Clinical symptoms range from pauci-symptomatic states presenting fever, cough and fatigue, to more severe forms including acute respiratory distress syndrome (ARDS) and/or other critically severe conditions [5]. Mortality is higher in the elderly, when the disease is at an initial stage and the virus replicates. In some cases, this activity triggers a second aggressive phase in which hyper-inflammation may require immediate urgent hospitalisation [6]. Patients admitted with these characteristics show a 20-fold increased risk of death compared to non-severe cases [7].

Timely identification of COVID-19 patients at higher risk of severe complications may enable early hospitalisation, risk-tailored clinical treatment and optimal allocation of human and technical resources [8]. In these circumstances, a predictive risk tool may conveniently identify subjects requiring immediate attention, serving as a reference in the relative lack of evidence-based guidelines. A variety of methods have been used to identify subjects with a higher risk of target outcomes e.g. COVID-19 diagnosis, hospital admission and negative prognosis. However, their direct applicability in clinical practice appears still limited [9–11].

The triage of COVID-19 patients requires multiple parameters to be assessed by a multidisciplinary team at presentation to the Emergency Department (ED) [12]. Characteristics that need to be routinely evaluated include clinical signs and symptoms known to be associated with subsequent prognosis [9] and various laboratory measurements e.g. decrease in albumin and increase in C-reactive protein (CRP), lactate dehydrogenase (LDH), lymphopenia and other haematological parameters that have been less investigated in clinical settings [9, 13].

In the framework of everyday care provided during the first wave of the outbreak, our aim was to identify patients diagnosed with COVID-19 with a higher risk of four key outcomes: hospital admission, mechanical ventilation, admission to Intensive Care Unit (ICU) and death.

The present study is a retrospective review of all consecutive cases presented at the ED over 3 months, addressing the following key research questions:

- Which demographic, clinical and laboratory parameters are associated with a higher risk for each of the key outcomes identified?
- How accurate are predictive models using only characteristics available at presentation to the ED?

## Materials and methods

### Study population

We performed a retrospective analysis of all consecutive patients presented with a confirmed diagnosis of COVID-19 at the ED of the General Hospital of Pescara (Abruzzo, Italy), between 1st march – 30th June 2020. The diagnosis was confirmed through a swab test performed by the hospital personnel in the same occasion. The time frame follows the start of the outbreak of COVID-19 in Italy [14]. At that time, the province of Pescara was the province most affected in the macro-area of Central/Southern Italy (+ 15.6% excess deaths compared to previous years) [15].

The Pescara General Hospital (PGH) is an urban 650-bedded tertiary facility of regional reference for adult traumas, acute diseases of neurosurgical interest, and COVID-19. It includes two ICUs: an 11-bedded facility, receiving critically ill patients from other EDs in the region and most of the other wards in the same hospital, and a 24-bedded COVID Unit, specifically opened to respond to the emergency of the pandemic.

Data were merged from records available at different sources, including the hospital discharge abstract database, the computerized hospital information system including personal health records of laboratory measurements, and paper-based clinical abstracts, from which other characteristics were manually extracted.

Two additional samples were collected for external validation, using a large subset of entries to the same ED between December 2020–January 2021 (corresponding to the transition from the “Wild” lineage of COVID-19 to the “Alpha” variant) [16] and January–March 2022 (covering the transition between the “Delta” variant and “Omicron”) [17]. All data were included in an Excel sheet accessed for statistical analysis.

The study was conducted according to the Declaration of Helsinki (amended version). The local Health Administrative Board reviewed and approved the study plan submitted by the Infectious Diseases Unit, the Emergency Department and the Laboratory Staff of PGH. The use of anonymised clinical and laboratory data for institutional research purposes was granted through signed informed consent upon hospital admission by all patients included in the study. Specific consent for the conduction of the study was not considered required, as confidentiality was guaranteed, and no specific interventions were performed beyond the ordinary good standard clinical practices.

#### Target characteristics

The study targeted four different outcomes after presentation to the ED: hospitalisation, oxygen support, admission to the ICU, and death (in or out of hospital). By definition, not all combinations were possible, e.g. a patient can be discharged at home and experience a fatal event or can progress through all transitions from hospitalisation. Some states directly implied others e.g. a patient receiving oxygen support or admitted to the ICU must be hospitalised.

Demographic and clinical characteristics, signs and symptoms and laboratory measurements were considered as potential predictors for the selected outcomes.

Age and gender were used as the only demographic characteristics of interest. For clinical variables, we considered a history of six comorbid conditions: diabetes, cardiovascular diseases (CVD), obesity, cancer, End Stage Renal Disease (ESRD), chronic obstructive pulmonary disease (COPD) and hypertension. These characteristics were recorded as part of routine clinical practice, using standardized definitions adopted by all hospitals in Italy.

Signs and symptoms related to COVID-19 were also considered, e.g. fever, cough, asthenia, diarrhea and dyspnea. Accurate recording was ensured by a standard national protocol applied routinely to monitor patients diagnosed with COVID-19.

Finally, eight device-assisted and laboratory parameters were considered as potential predictors of patients’ prognosis, based on high/low levels considered as potential correlates to the severity of the disease [18]: procalcitonin, lactate dehydrogenase (LDH), monocyte distribution width (MDW), oxygen saturation level, D-dimer, prothrombin time, C-reactive Protein (CRP) and lymphocyte counts. Among these, only the levels of procalcitonin were pre-assigned, based on a threshold considered valid for all outcomes (value = 0.2 ng/mL). The remaining seven parameters were transformed into binary variables by using optimal thresholds for each of the four outcomes, defined by the maximum Youden index in a univariate ROC analysis [19]. The procedure allowed identifying a set of low/high levels for measures without prior targeted cut-offs for COVID-19-related outcomes (see Table 1).

**Table 1:** Categorization of continuous variables identified by ROC analyses (max Youden index)

Variable	Category	Hospitalisation	Oxygen Support	Intensive Therapy	Death
		Cutoff	Cutoff	Cutoff	Cutoff
**LDH U/L**	Low	< 240	< 237	< 334	< 307
	High	≥240	≥237	≥334	≥307
**MDW**	Low	< 22	< 24	< 25	< 26
	High	≥22	≥24	≥25	≥26
**Saturation %**	Low	< 96	< 95	< 92	< 96
	High	≥ 96	≥95	≥92	≥96
**D-dimer mg/L**	Low	< 0.72	< 0.55	< 2.37	< 1.04
	High	≥0.72	≥0.55	≥2.37	≥1.04
**Prothrombin time %**	Low	< 95	< 88	< 85	< 77
	High	≥95	≥88	≥85	≥77
**CRP mg/L**	Low	< 21	< 42	< 68	< 43
	High	≥21	≥42	≥68	≥43
**Lymphocytes x10**^**3**^**μL**	Low	< 1	< 1.2	< 0.7	< 0.7
	High	≥1	≥1.2	≥0.7	≥0.7

#### Statistical analysis

We investigated hospitalisation and death in/out of hospital through follow-up of all patients in the study population. Oxygen support and admission to intensive care included only those hospitalised with less than 70 years, to avoid potential bias of deaths occurring before either option as a competing risk [20]. The choice is consistent with the guideline of avoiding transfer to ICU for patients aged >75y. During the reference timeframe, only 3 patients died in the selected subgroup.

Descriptive analysis included the calculation of mean and standard deviation for continuous variables and absolute and relative frequencies for categorical variables. Optimal thresholds for hospitalisation and oxygen support were used to calculate frequencies for the reference population in the overall sample and among those aged<70y hospitalised. Logistic regression was used for univariate and multivariate odds ratios (OR) of non-fatal outcomes [21]. Survival analysis was used to consider censoring in the analysis of time to fatal events, calculated as the difference between the date of death and admission to the hospital. For survivors, the censoring time was defined as the lag between presentation at the ED and the earliest date between the first negative swab result (an indicator of full recovery) and the date of study closure (30th June 2020). Cox proportional hazards was used for the calculation of univariate and multivariate hazard ratios (HR) for time-to-event analysis [22]. An alpha level of 0.05 was used to present odds and hazard ratios together with their 95% confidence intervals (95%CI) and *p* values. Forest plots were used to visualize results.

Predictive factors using a fully automated four-step backward elimination process in all multivariate regressions. Age and gender were forced in all models, with all other variables sequentially excluded in three consecutive rounds using a *p* value ≥0.20, ≥0.10 and ≥ 0.05.

Predictive formulas were defined using the regression coefficients as follows [23]:

- odds/hazard ratios < 1 were turned into their reciprocal value and assigned a negative sign;
- odds/hazard ratios greater or equal to 1.5 were rounded to their next integer value (to reflect increased risk by a multiplicative factor);
- odds/hazard ratios between 1 and 1.5 were transformed into their difference from 1 (rounded to the second decimal, to reflect increased risk by a percentage).

Total risk scores for each patient in the database were computed separately for each outcome, adding up all coefficients indicated above for all significant variables. A ROC analysis was performed using a separate 2 × 2 “confusion matrix” for every possible threshold applied to the total score [24]. The best threshold was defined by the highest value of the Youden Index [19]. Point estimates and confidence intervals were computed using the bootstrap for sensitivity, specificity, Positive Predictive Value (PPV), Negative Predictive Value (NPV) and the De Long method for the Area Under the Curve (AUC) [25].

The same measures were produced for both the study sample and the additional datasets collected for external validation. Survival results for validation samples were only available as odds ratios, as Cox proportional hazards was not applicable due to the unavailability of time of negative swab test.

Reliability analysis of the predictive formula was summarized using ROC curves showing optimal thresholds and sensitivity, specificity, AUC with point estimates and 95% confidence intervals. All statistical analyses were carried out by developing ad hoc software in the R language [26].

## Results

A total of 536 consecutive admissions with a positive molecular assay to SARS-CoV2 were recorded at the ED in the reference period. The samples collected for validation were equal to 224 out of 639 (35%) for December 2020 – January 2021 and 375 out of a total of 872 (43%) for January–March 2022.

Among subjects hospitalised in the baseline study sample, a total of 174 consecutive admissions involved patients aged less than 70 years. The samples used for validation were equal to 95 out of 226 (42%) for December 2020 – January 2021 and 56 out of a total of 135 (41%) for January–March 2022.

All details of the association including risk ratios and 95% confidence intervals for the total population admitted to the ED at baseline and those hospitalised below 70 are reported in Tables 2 and 3 respectively.

**Table 2:** Descriptive frequencies for the overall sample of patients admitted to the Emergency Department ^a^

Variable	Category	Reference Population	Hospitalisation				Death			
			No	Yes	OR (95% CI)	***P***>𝛘^**2**^	No	Yes	HR (95% CI)	***P***>𝛘^**2**^
**N**	**N.Obs**	**536 (100.0)**	**171 (31.9)**	**365 (68.1)**			**400 (74.6)**	**136 (25.4)**		
**Age**^**b**^	Continuous	63.2 (19.1)	50.4 (17.7)	69.2 (16.7)	1.06 (1.05–1.08)	< 0.001	57.4 (17.2)	80.4 (13.2)	1.08 (1.06–1.09)	< 0.001
**Gender**	Female	264 (49.3)	99 (57.9)	165 (45.2)	1.00 (−)	–	201 (50.2)	63 (46.3)	1.00 (−)	–
	Male	272 (50.7)	72 (42.1)	200 (54.8)	1.67 (1.15–2.41)	< 0.01	199 (49.8)	73 (53.7)	1.12 (0.80–1.57)	0.516
**Diabetes**	No	449 (83.8)	164 (95.9)	285 (78.1)	1.00 (−)	–	348 (87.0)	101 (74.3)	1.00 (−)	–
	Yes	86 (16.0)	7 (4.1)	79 (21.6)	6.49 (2.93–14.40)	< 0.001	52 (13.0)	34 (25.0)	1.87 (1.27–2.76)	< 0.01
	Missing	1 (0.2)	–	1 (0.3)	–	–	–	1 (0.7)	–	–
**CVD**	No	396 (73.9)	148 (86.5)	248 (67.9)	1.00 (−)	–	329 (82.2)	67 (49.3)	1.00 (−)	–
	Yes	139 (25.9)	23 (13.5)	116 (31.8)	3.01 (1.84–4.92)	< 0.001	71 (17.8)	68 (50.0)	3.50 (2.49–4.90)	< 0.001
	Missing	1 (0.2)	–	1 (0.3)	–	–	–	1 (0.7)	–	–
**Obesity**	No	479 (89.4)	167 (97.7)	312 (85.5)	1.00 (−)	–	361 (90.2)	118 (86.8)	1.00 (−)	–
	Yes	27 (5.0)	1 (0.6)	26 (7.1)	13.92 (1.87–103.47)	< 0.001	21 (5.2)	6 (4.4)	0.92 (0.40–2.08)	0.830
	Missing	30 (5.6)	3 (1.8)	27 (7.4)	–	–	18 (4.5)	12 (8.8)	–	–
**Cancer**	No	490 (91.4)	164 (95.9)	326 (89.3)	1.00 (−)	–	369 (92.2)	121 (89.0)	1.00 (−)	–
	Yes	46 (9.6)	7 (4.1)	39 (10.7)	2.80 (1.23–6.40)	< 0.001	31 (7.8)	15 (11.0)	1.41 (0.83–2.41)	0.228
**ESRD**	No	509 (95.0)	170 (99.4)	339 (92.9)	1.00 (−)	–	394 (98.5)	115 (84.6)	1.00 (−)	
	Yes	26 (4.8)	1 (0.6)	25 (6.8)	12.54 (1.68–93.31)	< 0.001	6 (1.5)	20 (14.7)	5.17 (3.21–8.34)	< 0.001
	Missing	1 (0.2)	–	1 (0.3)	–	–	–	1 (0.7)	–	–
**COPD**	No	490 (91.4)	166 (97.1)	324 (88.8)	1.00 (−)	–	379 (94.8)	111 (81.6)	1.00 (−)	–
	Yes	46 (8.6)	5 (2.9)	41 (11.2)	4.20 (1.63–10.83)	< 0.001	21 (5.2)	25 (18.4)	3.02 (1.95–4.66)	< 0.001
**Hypertension**	No	341 (63.6)	146 (85.4)	195 (53.4)	1.00 (−)	–	277 (69.2)	64 (47.1)	1.00 (−)	
	Yes	194 (36.2)	25 (14.6)	169 (46.3)	5.06 (3.16–8.11)	< 0.001	123 (30.8)	71 (52.2)	2.08 (1.48–2.91)	< 0.001
	Missing	1 (0.2)	–	1 (0.3)	–	–	–	1 (0.7)	–	–
**Fever**	No	108 (62.1)	56 (32.7)	52 (14.2)	1.00 (−)	–	77 (19.2)	31 (22.8)	1.00 (−)	–
	Yes	428 (79.9)	115 (67.3)	313 (85.8)	2.93 (1.90–4.52)	< 0.001	323 (80.8)	105 (77.2)	0.79 (0.53–1.18)	0.252
**Cough**	No	279 (52.0)	99 (57.9)	180 (49.3)	1.00 (−)	–	193 (48.2)	86 (63.2)	1.00 (−)	
	Yes	234 (43.7)	69 (40.4)	165 (45.2)	1.32 (0.91–1.91)	0.149	190 (47.5)	44 (32.4)	0.53 (0.37–0.76)	< 0.001
	Missing	23 (4.3)	3 (1.8)	20 (5.5)	–	–	17 (4.2)	6 (4.4)	–	–
**Asthenia**	No	391 (73.0)	125 (73.1)	266 (72.9)	1.00 (−)	–	280 (70.0)	111 (81.6)	1.00 (−)	
	Yes	95 (17.7)	30 (17.5)	65 (17.8)	1.02 (0.63–1.65)	0.942	81 (20.2)	14 (10.3)	0.47 (0.27–0.82)	< 0.01
	Missing	50 (9.3)	16 (9.4)	34 (9.3)	–	–	39 (9.8)	11 (8.1)	–	–
**Diarrhea**	No	68 (12.7)	32 (18.7)	36 (9.9)	1.00 (−)	–	336 (84.0)	131 (96.3)	1.00 (−)	
	Yes	467 (87.1)	139 (81.3)	328 (89.9)	0.48 (0.28–0.80)	< 0.01	64 (16.0)	4 (2.9)	0.20 (0.07–0.54)	< 0.001
	Missing	1 (0.2)	–	1 (0.3)	–	–	–	1 (0.7)	–	–
**Dyspnea**	No	303 (56.5)	136 (79.5)	167 (45.8)	1.00 (−)	–	254 (63.5)	49 (36.0)	1.00 (−)	
	Yes	232 (43.3)	35 (20.5)	197 (54.0)	4.58 (3.00–7.01)	< 0.001	146 (36.5)	86 (63.2)	2.55 (1.80–3.62)	< 0.001
	Missing	1 (0.2)	–	1 (0.3)	–	–	–	1 (0.7)	–	–
**Procalcitonin ng/mL**	< 0.2	370 (69.0)	155 (90.6)	215 (58.9)	1.00 (−)	–	325 (81.2)	45 (33.1)	1.00 (−)	
	≥0.2	159 (29.7)	9 (5.3)	150 (41.1)	12.02 (5.95–24.28)	< 0.001	68 (17.0)	91 (66.9)	6.30 (4.40–9.02)	< 0.001
	Missing	7 (1.3)	7 (4.1)	–	–	–	7 (1.8)	0 (0.0)	–	–
**LDH U/L**	Low	315 (58.8)	51 (29.8)	264 (72.3)	1.00 (−)	–	245 (61.3)	46 (33.8)	1.00 (−)	–
	High	214 (41.2)	113 (66.1)	101 (27.7)	5.79 (3.87–8.66)	< 0.001	148 (37.0)	90 (66.2)	2.56 (1.80–3.66)	< 0.001
	Missing	–	7 (4.1)	–	–	–	7 (1.8)	–	–	–
**MDW**	Low	381 (71.1)	72 (42.1)	309 (84.7)	1.00 (−)	–	266 (66.5)	65 (47.8)	1.00 (−)	–
	High	151 (28.2)	98 (57.3)	53 (14.5)	7.94 (5.21–12.09)	< 0.001	132 (33.0)	69 (50.7)	1.76 (1.25–2.47)	< 0.01
	Missing	4 (0.7)	1 (0.6)	3 (0.8)	–	–	2 (0.5)	2 (1.5)	–	–
**Saturation %**	High	284 (53.0)	148 (86.5)	136 (37.3)	1.00 (−)	–	255 (63.7)	29 (21.3)	1.00 (−)	
	Low	247 (46.1)	21 (12.3)	226 (61.9)	11.71 (7.07–19.39)	< 0.001	141 (35.2)	106 (77.9)	4.83 (3.20–7.28)	< 0.001
	Missing	5 (0.9)	2 (1.2)	3 (0.8)	–	–	4 (1.0)	1 (0.7)	–	–
**D-dimer mg/L**	Low	289 (53.9)	36 (21.1)	253 (69.3)	1.00 (−)	–	282 (70.5)	35 (25.7)	1.00 (−)	–
	High	232 (43.3)	121 (70.8)	111 (30.4)	7.66 (4.96–11.82)	< 0.001	104 (26.0)	100 (73.5)	5.71 (3.88–8.39)	< 0.001
	Missing	15 (2.8)	14 (8.2)	1 (0.3)	–	–	14 (3.5)	1 (0.7)	–	–
**Prothrombin Time %**	High	165 (30.8)	87 (50.9)	78 (21.4)	1.00 (−)	–	324 (81.0)	86 (63.2)	1.00 (−)	–
	Low	367 (68.5)	80 (46.8)	287 (78.6)	4.00 (2.70–5.93)	< 0.001	72 (18.0)	50 (36.8)	2.17 (1.53–3.07)	< 0.001
	Missing	4 (0.7)	4 (2.3)	0 (0.0)	–	–	4 (1.0)	0 (0.0)	–	–
**CRP mg/L**	Low	336 (62.7)	40 (23.4)	296 (81.1)	1.00 (−)	–	237 (59.2)	33 (24.3)	1.00 (−)	–
	High	200 (37.3)	131 (76.6)	69 (18.9)	14.05 (9.04–21.82)	< 0.001	163 (40.8)	103 (75.7)	3.64 (2.46–5.40)	< 0.001
**Lymphocytes x10**^**3**^**μL**	High	297 (55.4)	137 (80.1)	160 (43.8)	1.00 (−)	–	313 (78.2)	70 (51.5)	1.00 (−)	
	Low	239 (44.6)	34 (19.9)	205 (56.2)	5.16 (3.36–7.93)	< 0.001	87 (21.8)	66 (48.5)	2.71 (1.93–3.79)	< 0.001

^a^Numbers in table are N (%); ^b^Mean (standard deviation)

**Table 3:** Descriptive frequencies for the sample of patients hospitalised with age less than 70 years^a^

Variable	Category	Hospitalised Population Age < 70	Oxygen Support				Intensive Therapy			
			No	Yes	OR (95% CI)	***P***>𝛘^**2**^	No	Yes	OR (95% CI)	***P***>𝛘^**2**^
**N**	**N.Obs**	**174 (100.0)**	**45 (25.9)**	**129 (74.1)**			**123 (70.7)**	**51 (29.3)**		
**Age**^**b**^	Continuous	54.3 (10.1)	51.4 (10.6)	55.4 (9.8)	1.04 (1.00–1.07)	0.250	53.1 (10.1)	57.2 (9.4)	1.05 (1.01–1.09)	0.010
**Gender**	Female	62 (35.6)	17 (37.8)	45 (34.9)	1.00 (−)	–	46 (37.4)	16 (31.4)	1.00 (−)	–
	Male	112 (64.4)	28 (62.2)	84 (65.1)	1.13 (0.56–2.29)	0.728	77 (62.6)	35 (68.6)	1.31 (0.65–2.62)	0.447
**Diabetes**	No	142 (81.6)	38 (84.4)	104 (80.6)	1.00 (−)	–	106 (86.2)	36 (70.6)	–	–
	Yes	32 (18.4)	7 (15.6)	25 (19.4)	1.30 (0.52–3.26)	0.563	17 (13.8)	15 (29.4)	0.78 (0.27–2.28)	0.648
	Missing	–	–	–	–	–	–	–	–	–
**CVD**	No	154 (88.5)	40 (88.9)	114 (88.4)	1.00 (−)	–	108 (87.8)	46 (90.2)	–	–
	Yes	20 (11.5)	5 (11.1)	15 (11.6)	1.05 (0.36–3.08)	0.925	15 (12.2)	5 (9.8)	3.50 (2.49–4.90)	< 0.001
	Missing	–	–	–	–	–	–	–	–	–
**Obesity**	No	142 (81.6)	37 (82.2)	105 (81.4)	1.00 (−)	–	104 (84.6)	38 (74.5)	1.00 (−)	
	Yes	16 (9.2)	4 (8.9)	12 (9.3)	1.06 (0.32–3.48)	0.927	11 (8.9)	5 (9.8)	1.24 (0.41–3.81)	0.706
	Missing	16 (9.2)	4 (8.9)	12 (9.3)	–	–	8 (6.5)	8 (15.7)	–	–
**Cancer**	No	164 (94.3)	40 (88.9)	124 (96.1)	1.00 (−)	–	116 (94.3)	48 (94.1)	1.00 (−)	–
	Yes	10 (5.7)	5 (11.1)	5 (3.9)	0.32 (0.09–1.17)	0.920	7 (5.7)	3 (5.9)	1.04 (0.26–4.17)	0.961
**ESRD**	No	171 (98.3)	45 (100.0)	126 (97.7)	1.00 (−)	–	122 (99.2)	49 (96.1)	1.00 (−)	
	Yes	3 (1.7)	–	3 (2.3)	2.52 (0.13–49.70)	0.178	1 (0.8)	2 (3.9)	4.98 (0.44–56.18)	0.177
	Missing	–	–	–	–	–	–	–	–	–
**COPD**	No	165 (94.8)	44 (97.8)	121 (93.8)	1.00 (−)	–	118 (95.9)	47 (92.2)	1.00 (−)	
	Yes	1 (5.2)	1 (2.2)	8 (6.2)	2.91 (0.35–23.93)	0.260	5 (4.1)	4 (7.8)	2.01 (0.52–7.81)	0.323
**Hypertension**	No	115 (66.1)	33 (73.3)	82 (63.6)	1.00 (−)	–	83 (67.5)	32 (62.7)	1.00 (−)	
	Yes	59 (33.9)	12 (26.7)	47 (36.4)	1.58 (0.74–3.34)	0.227	40 (32.5)	19 (37.3)	1.23 (0.62–2.44)	0.550
	Missing	–	–	–	–	–	–	–	–	–
**Fever**	No	13 (7.5)	6 (13.3)	7 (5.4)	1.00 (−)	–	8 (6.5)	5 (9.8)	1.00 (−)	–
	Yes	161 (92.5)	39 (86.7)	122 (94.6)	2.68 (0.85–8.46)	0.100	115 (93.5)	46 (90.2)	0.64 (0.20–2.06)	0.462
**Cough**	No	59 (33.9)	20 (44.4)	39 (30.2)	1.00 (−)	–	41 (33.3)	18 (35.3)	1.00 (−)	
	Yes	106 (60.9)	22 (48.9)	84 (65.1)	1.96 (0.96–4.00)	0.660	76 (61.8)	30 (58.8)	0.90 (0.45–1.81)	0.765
	Missing	9 (5.2)	3 (6.7)	6 (4.7)	–	–	6 (4.9)	3 (5.9)	–	–
**Asthenia**	No	113 (64.9)	28 (62.2)	85 (65.9)	1.00 (−)	–	79 (64.2)	34 (66.7)	1.00 (−)	
	Yes	44 (25.3)	10 (22.2)	34 (26.4)	1.12 (0.49–2.55)	0.787	31 (25.2)	13 (25.5)	0.97 (0.45–2.09)	0.947
	Missing	7 (9.8)	7 (15.6)	10 (7.8)	–	–	13 (10.6)	4 (7.8)	–	–
**Diarrhea**	No	148 (85.1)	37 (82.2)	111 (86.0)	1.00 (−)	–	103 (83.7)	45 (88.2)	1.00 (−)	
	Yes	26 (14.9)	8 (17.8)	18 (14.0)	0.75 (0.30–1.87)	0.542	20 (16.3)	6 (11.8)	0.69 (0.26–1.82)	0.440
	Missing	–	–	–	–	–	–	–	–	–
**Dyspnea**	No	87 (50.0)	29 (64.4)	58 (45.0)	1.00 (−)	–	66 (53.7)	21 (41.2)	1.00 (−)	
	Yes	87 (50.0)	16 (35.6)	71 (55.0)	2.22 (1.10–4.48)	0.240	57 (46.3)	30 (58.8)	1.65 (0.85–3.20)	0.133
	Missing	–	–	–	–	–	–	–	–	–
**Procalcitonin ng/mL**	< 0.2	131 (75.3)	40 (88.9)	91 (70.5)	1.00 (−)		98 (79.7)	33 (64.7)	1.00 (−)	
	≥0.2	43 (24.7)	5 (11.1)	38 (29.5)	3.34 (1.22–9.12)	< 0.01	25 (20.3)	18 (35.3)	2.14 (1.04–4.41)	0.410
	Missing	–	–	–	–	–	–	–	–	–
**LDH U/L**	Low	44 (25.3)	26 (57.8)	18 (14.0)	1.00 (−)	–	75 (61.0)	11 (21.6)	1.00 (−)	–
	High	130 (74.7)	19 (42.2)	111 (86.0)	8.44 (3.89–18.29)	< 0.001	48 (39.0)	40 (78.4)	5.68 (2.66–12.14)	< 0.001
	Missing	–	–	–	–	–	–	–	–	–
**MDW**	Low	57 (32.8)	26 (57.8)	31 (24.0)	1.00 (−)	–	64 (52.0)	12 (23.5)	1.00 (−)	–
	High	117 (67.2)	19 (42.2)	98 (76.0)	4.33 (2.11–8.85)	< 0.001	59 (48.0)	39 (76.5)	3.53 (1.69–7.37)	< 0.001
	Missing	–	–	–	–	–	–	–	–	–
**Saturation %**	High	91 (52.3)	40 (88.9)	51 (39.5)	1.00 (−)		103 (83.7)	26 (51.0)	1.00 (−)	
	Low	81 (47.7)	4 (8.9)	77 (59.7)	15.10 (5.09–44.77)	< 0.001	19 (15.4)	24 (47.1)	5.00 (2.39–10.49)	< 0.001
	Missing	–	1 (2.2)	1 (0.8)	–	–	1 (0.8)	1 (2.0)	–	–
**D-dimer mg/L**	Low	61 (35.1)	18 (40.0)	43 (33.3)	1.00 (−)	–	119 (96.7)	45 (88.2)	1.00 (−)	–
	High	113 (64.9)	27 (60.0)	86 (66.7)	1.33 (0.66–2.68)	0.423	4 (3.3)	6 (11.8)	3.97 (1.07–14.71)	0.380
	Missing	–	–	–	–	–	–	–	–	–
**Prothrombin Time %**	High	77 (44.3)	22 (48.9)	55 (42.6)	1.00 (−)		74 (60.2)	24 (47.1)	1.00 (−)	
	Low	97 (55.7)	23 (51.1)	74 (57.4)	1.29 (0.65–2.54)	0.468	49 (39.8)	27 (52.9)	1.70 (0.88–3.28)	0.114
	Missing	–	0 (0.0)	0 (0.0)	–	–	0 (0.0)	0 (0.0)	–	–
**CRP mg/L**	Low	65 (37.4)	28 (62.2)	37 (28.7)	1.00 (−)	–	76 (61.8)	17 (33.3)	1.00 (−)	–
	High	109 (62.6)	17 (37.8)	92 (71.3)	4.10 (2.01–8.36)	< 0.001	47 (38.2)	34 (66.7)	3.23 (1.63–6.43)	< 0.001
**Lymphocytes x10**^**3**^**μL**	High	56 (32.2)	25 (55.6)	31 (24.0)	1.00 (−)	–	90 (73.2)	26 (51.0)	1.00 (−)	
	Low	118 (67.8)	20 (44.4)	98 (76.0)	3.95 (1.94–8.06)	< 0.001	33 (26.8)	25 (49.0)	2.62 (1.33–5.17)	< 0.01

^a^Numbers in table are N (%); ^b^Mean (standard deviation)

### Hospitalisation and deaths (in or out of hospital)

The mean age (standard deviation) was 63.2 (19.1) y, with 50.7% of males. The most frequent comorbidities were hypertension (36.2%), CVD (25.9%) and diabetes (16.0%). The most frequent symptom was diarrhea (87.1%), followed by fever (79.9%), cough (43.7%) and dyspnea (43.3%). The parameter that was most frequently abnormal was prothrombin time < 95% (68.5%), followed by saturation < 96% (46.1%), lymphocytes< 1 × 10^3^/μL (44.6%), D-dimer≥0.72 mg/L (43.3%), LDH ≥ 240 U/L (41.2%), CRP ≥ 21 mg/L (37.3%) and MDW ≥ 22 (28.2%).

A total of 365 subjects (68.1%) were hospitalised after presentation to the ED. Among the main baseline characteristics observed, only cough and asthenia were not significantly associated with hospitalisation. An additional year of age was associated with a 6% increased risk of hospitalisation, while males were almost 70% more likely to be hospitalised. An over ten fold association with increased risk was found for CRP ≥ 21 mg/L, obesity, ESRD and saturation < 96%. Associated risk was over five fold for MDW ≥ 22, D-dimer ≥0.72 mg/L, diabetes, hypertension, LDH ≥ 240 U/L and lymphocytes< 1 × 10^3^/μL. Moderate association was found for dyspnea, COPD, CVD, cough and cancer. Patients with diarrhea were over 50% less likely to be hospitalised.

A total of 136 subjects (25.4%) died in or out of hospital in the reference timeframe, following presentation to the ED with a confirmed diagnosis of COVID-19. Among the main characteristics, gender, obesity, fever and cancer were not significantly associated with survival. An additional year of age was associated with a 8% increased risk of death, and an over threefold increase for procalcitonin≥0.2 ng/mL, D-dimer≥1.04 mg/L, ESRD, saturation < 96%, CVD, CRP ≥ 43 mg/L and COPD. Risk was more than doubled for lymphocytes< 0.7 × 10^3^/μL, LDH ≥ 307 U/L, dyspnea, prothrombin time < 77% and hypertension. Moderate increased risk was found for diabetes and MDW. On the other hand, three conditions were found to be associated with survival after diagnosis with COVID-19: patients with diarrhea were 80% less likely to die, with almost a 50% risk reduction also observed for those presented with asthenia and cough.

### Oxygen support and intensive care

The mean age (standard deviation) was 54.3 (10.1) y, with 64.4% of males. The most frequent comorbidities were hypertension (33.9%) and diabetes (18.4%). The most frequent symptoms were fever (92.5%) and cough (60.9%). The parameter found most frequently abnormal was LDH ≥ 237 U/L (74.7%), followed by lymphocytes < 1.2 × 10^3^/μL (67.8%), MDW ≥ 24 (67.2%), D-dimer≥0.55 mg/L (64.9%), CRP ≥ 42 mg/L (62.6%) and prothrombin time < 88% (55.7%). Less than half of the patients had a saturation < 95% (47.7%) and procalcitonin ≥0.2 ng/mL (24.7%).

A total of 129 subjects (74.1%) received oxygen after hospitalisation. None of the demographic and clinical characteristics were significantly associated with such treatment. Among relevant parameters, a level of increased risk between four and fifteen-fold was found for saturation < 95%, LDH ≥ 237 U/L, MDW ≥ 24, CRP ≥ 42 mg/L and lymphocytes< 1.2 × 10^3^/μL. Over three-fold association was found for procalcitonin≥0.2 ng/mL. D-dimer and prothrombin time were not associated with oxygen support.

A total of 51 subjects (29.3%) aged below 70 were admitted to the ICU after hospitalisation. Among subject characteristics, only age and CVD were associated with increased risk of intensive care. Among laboratory and device-assisted measurements, risk was at least five times higher for LDH ≥ 334 U/L and saturation < 92%, more than tripled for MDW ≥ 25 and CRP ≥ 68 mg/L, more than doubled for lymphocytes< 0.7 × 10^3^/μL. Procalcitonin, D-dimer and prothrombin time were not associated with admission to ICU.

### Multivariate analysis

**Fig. 1:**
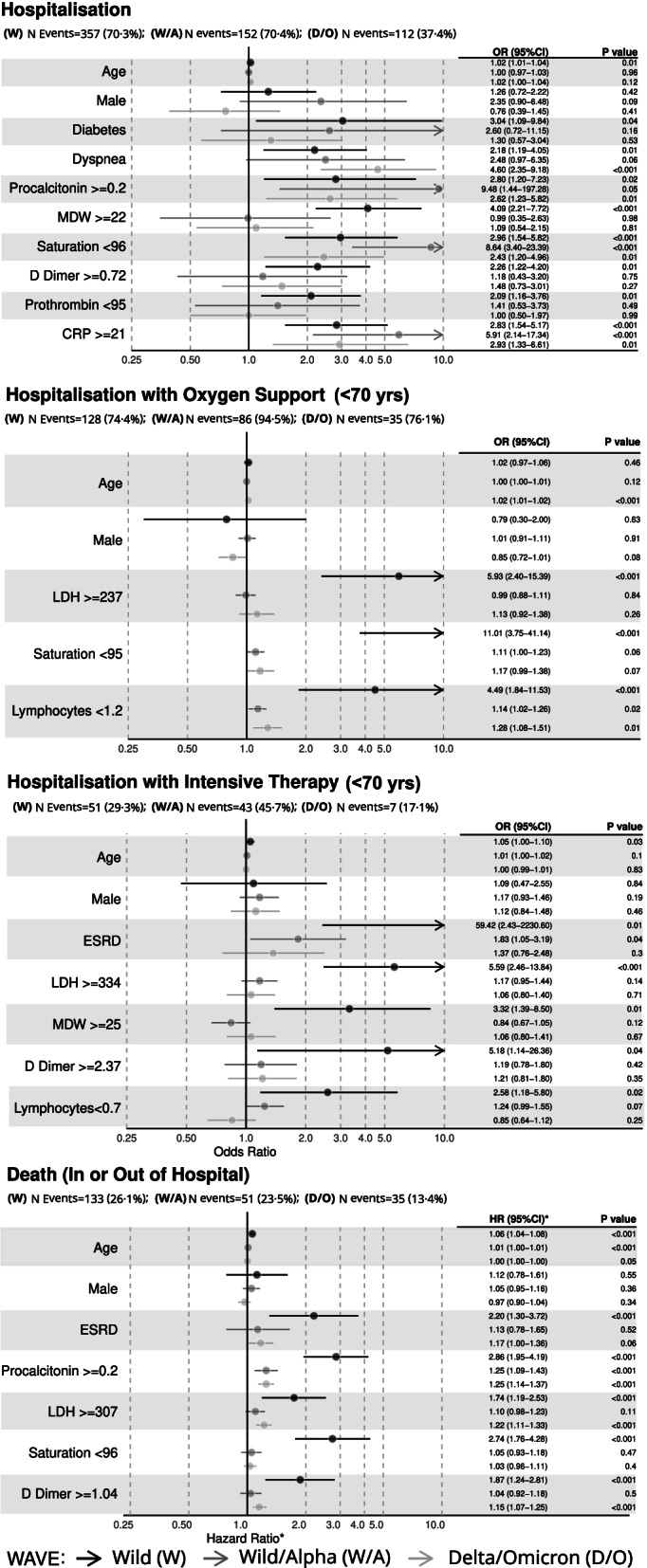
Forest plot showing results of multivariate analysis for multiple outcomes and validation periods (* Odds Ratios computed for validation samples)

The results of multivariate analysis are shown along with those obtained from validation over different samples in Fig. 1.

Among adjustment terms, only age (unit increase) was associated with a higher risk of experiencing three of the four outcomes of interest, namely hospitalisation (OR = 1.02; 1.01–1.04), intensive care (OR = 1.05; 1.00–1.10) and death (HR = 1.06; 1.04–1.08).

Taking into account all potential confounders, further eight characteristics were significantly associated to an increased risk of hospitalisation. The risk was over four fold for patients with MDW ≥ 22 (OR = 4.09; 2.21–7.72), and over three fold for people with diabetes (OR = 3.04; 1.09–9.84). All other significant characteristics had a risk between two and three times higher than their reference category: saturation < 96% (OR = 2.96; 1.54–5.82), CRP ≥ 21 mg/L (OR = 2.83; 1.54–5.17), procalcitonin≥0.2 ng/mL (OR = 2.80; 1.20–7.23), D-dimer≥0.72 mg/L (OR = 2.26; 1.22–4.20), dyspnea (OR = 2.18; 1.19–4.05) and prothrombin time < 95% (OR = 2.09; 1.16–3.76).

Only one clinical characteristic was associated with an increased risk of death: ESRD (HR = 2.20; 1.30–3.72). Other four categories with abnormal levels were significantly associated with an increased risk: procalcitonin≥0.2 ng/mL (HR = 2.86; 1.95–4.19), saturation < 96% (HR = 2.74; 1.76–4.28), D-dimer≥1.04 mg/L (HR = 1.87; 1.24–2.81) and LDH ≥ 307 U/L (HR = 1.74; 1.19–2.53).

After hospitalisation, oxygen support was over ten times more likely for saturation < 95% (OR = 11.01; 3.75–41.14), almost six times higher for LDH ≥ 237 U/L (OR = 5.93; 2.40–15.39) and over four times higher for lymphocytes< 1.2 × 10^3^/μL (OR = 4.49; 1.84–11.53).

Admission to ICU recorded the highest level of association found for a clinical characteristic, being almost sixty times more likely for ESRD (OR = 59.42; 2.43–2230.60). Other independent risk factors were LDH ≥ 334 U/L (OR = 5.59; 2.46–13.84), D-dimer≥2.37 mg/L (OR = 5.18; 1.14–26.36), MDW ≥ 25 (OR = 3.32; 1.39–8.50) and lymphocytes< 0.7 × 10^3^/μL (OR = 2.58; 1.18–5.80).

Several variables among those presented above were not significant in the validation over the extended timeframe.

### Risk scores

Risk scores were directly derived from predictive models, according to the method outlined above. The predictive accuracy of the algorithms is presented in Table 4. The validation results are available as Supplementary Data.

**Table 4:** Algorithms for the calculation of scores

	Hospitalisation				Oxygen Support < 70 yrs				Intensive Therapy < 70 yrs				Death			
**Algorithm** **Condition value:** **[1 = Yes; 0 = No]**	round(0.02 * [age]) + + 1 * [Male] + 3 * [Diabetes] + 2 * [Dyspnea] + 3 * [Procalcitonin **≥**0.2 ng/mL] + 4 * [MDW **≥** 22] + 3 * [Saturation ≤ 95%] + 2 * [D-dimer **≥**0.72 mg/L] + 2 * [Prothrombin time **<** 95%] + 3 * [CRP **≥** 21 mg/L]				6 * [LDH **≥**237 U/L] + 11 * [Saturation < 95%] + 4 * [Lymphocytes < 1.2 × 10^3^/μL]				round(0.05 * [Age]) + + 59 * [ESRD] + 6 * [LDH **≥** 334 U/L] + 3 * [MDW **≥** 25] + 5 * [D-dimer **≥**2.37 mg/L] + 3 * [Lymphocytes < 0.7 × 10^3^/μL]				round(0.06* [Age]) + + 2* [ESRD]) + 3* [Procalcitonin **≥**0.2 ng/mL]) + 2* [LDH **≥** 307 U/L]) + 3* [Saturation < 96%]) + 2* [D-dimer **≥**1.04 mg/L])			
**Optimal Cutpoint**	**≥12**				**≥8**				**≥10**				**≥10**			
**Study population (“Alpha”: May–June 2020)**																
**Confusion Matrix**		Yes	No			Yes	No			Yes	No			Yes	No	
	Score +	284	19	303	Score +	111	10	121	Score +	42	35	77	Score +	118	80	192
	Score -	73	132	205	Score -	17	34	51	Score -	9	88	97	Score -	15	297	312
	Total	357	151	508	Total	128	44	172	Total	51	126	174	Total	133	377	510
**Youden Index**	0.67				0.64				0.54				0.67			
**Sensitivity**	**0.80 (0.75–0.83)**				**0.87 (0.80–0.91)**				**0.82 (0.70–0.90)**				**0.89 (0.82–0.93)**			
**Specificity**	**0.87 (0.81–0.92)**				**0.77 (0.63–0.87)**				**0.71 (0.63–0.79)**				**0.79 (0.74–0.83)**			
**PPV**	0.94 (0.90–0.96)				0.92 (0.86–0.95)				0.54 (0.43–0.65)				0.60 (0.53–0.66)			
**NPV**	0.64 (0.58–0.71)				0.67 (0.53–0.78)				0.91 (0.83–0.95)				0.95 (0.92–0.97)			
**LR+**	6.32 (4.29–10.30)				3.82 (2.38–7.68)				2.89 (2.16–4.05)				4.18 (3.43–5.20)			
**LR-**	0.23 (0.19–0.29)				0.17 (0.10–0.26)				0.25 (0.11–0.41)				0.14 (0.08–0.22)			
**Accuracy**	0.82				0.84				0.75				0.81			
**AUC**	**0.91 (0.89–0.94)**				**0.87 (0.82–0.92)**				**0.81 (0.73–0.89)**				**0.91 (0.87–0.94)**			

For hospitalisation, an optimal cut point equal to 12 achieved sensitivity of 80% (95%CI: 75–83%) and specificity of 87% (81–92%). The overall performance was very high (AUC = 91%; 89–94%), suggesting that the predictive model may be suitable for regular use at ED.

The same performance was achieved with a cut-off equal to 10 by the mortality predictive model (AUC = 91%; 87–94%), with a higher sensitivity (89%; 82–93%), but lower specificity (79%; 74–83%).

Similar levels were obtained using a cut-off of 8 for oxygen support, achieving a sensitivity of 87% (80–91%) and a specificity of 77% (63–87%), with a slightly lower overall performance (AUC = 87%; 82–92%).

Finally, admission to ICU recorded the lowest performance with a cut-off equal to 10 and a sensitivity = 82% (70–90%), specificity = 71% (63–79%), and AUC = 81% (73–89%).

The external validation denoted an optimism in the accuracy measured by the AUC ranging between 3 and 19%.

**Fig. 2:**
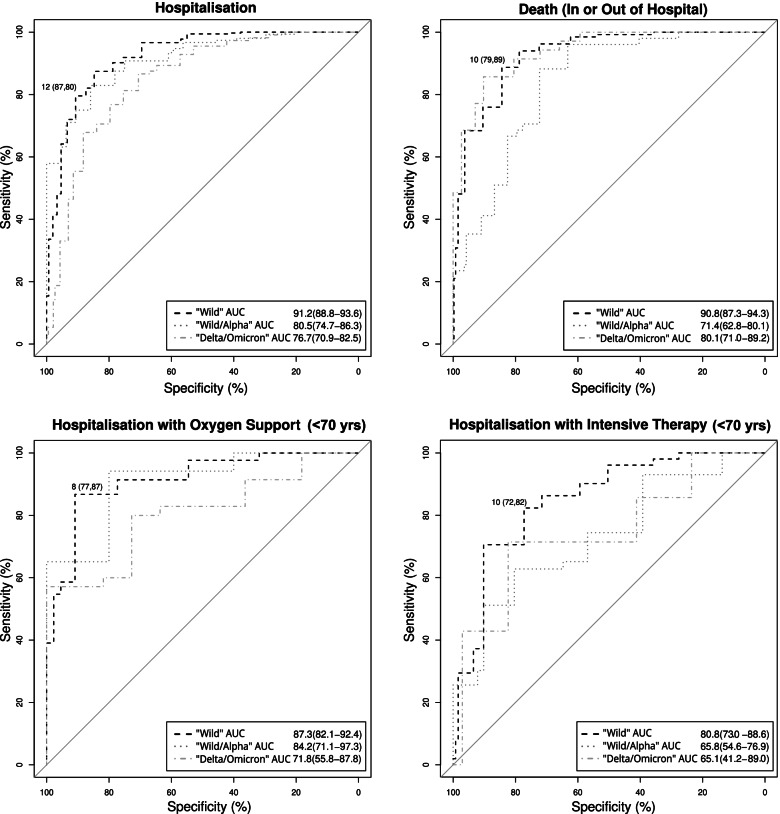
Overall ROC analysis showing the performances of algorithms over different validation samples

An overall ROC analysis of the performance achieved by all predictive models is presented in Fig. 2.

## Discussion

The Italian National Health System (Servizio Sanitario Nazionale, SSN) delivers national guidelines for the standard provision of health services across the country. However, when Italy was first hit by the coronavirus, hospitals experienced a rapid increase of hospitalisations, followed by an exceptional shortage of medical equipment and a limited set of recommendations regarding best practices [14]. The situation required immediate measures to organise local practices and provide urgent care. The rationale for the present study originates from the practical needs emerged during this phase of the emergency. To carry out our investigation, we strived to collect a large dataset that could help respond to the research questions posed at the outset.

Our results are consistent with those obtained by other studies carried out on different outcomes, including mortality, progression to severe/critical status, recovery, length of hospital stay, admission to ICU, intubation, duration of mechanical ventilation, acute distress respiratory, cardiac injury and thrombotic complications. Evidence showed predictive factors including age, comorbidities, vital signs, image features, gender, lymphocyte count and C-reactive protein [9].

In this study, we considered specific predictors, e.g. optimal cutoffs for MDW, which have not been previously considered by algorithms for COVID outcomes using data available during the early phase of admission, to support clinical decision in the Emergency Department.

We identified eight known targets of hospital management to be significantly associated with hospitalisation independently from age and gender: diabetes, dyspnea, procalcitonin≥0.2 ng/mL, MDW ≥ 22, saturation < 96%, D-dimer≥0.72 mg/L, prothrombin time < 95% and CRP ≥ 21 mg/L. Among them, MDW deserves to be presented in more detail. MDW is a novel haematological parameter recently introduced for the diagnosis of sepsis [18, 27], which has been already targeted by recent investigations [28]. Our study confirmed a significant association between MDW and sepsis [29–31], consistently with other viral diseases [29, 30, 32]. Changes in morphology and volumetric size of white blood cells are a well-documented consequence of cellular activation upon early infection, as a part of innate immunity response [33]. Monocytes are involved in the early response to infection, acting as first interceptors of the invading microorganism, for phagocytosis and further immune processing. Further studies showed changes in the morphology during inflammation [34], differentiating into amoeboid cells, as assessed by microscopy after Giemsa staining, and increased expression of functional markers such as CD16 [35]. Another study found that monocytes homeostasis and morphology appear considerably perturbed in patients with SARS-CoV2 infection [13], with MDW showing significantly elevated values also in patients with COVID-19, compared to other upper respiratory tract infections [36, 37]. As a consequence, the possibility of monitoring monocyte size in parallel with routine blood cell counts and other clinical indicators at presentation to the ED may represent a convenient tool to identify patients at high risk among those diagnosed with COVID-19.

As a first relevant transition after hospitalisation, we focused on oxygen support among patients with COVID-19 aged less than 70 years. In this group of subjects, dyspnea, chest distress and respiratory rate were found to be highly associated with oxygen therapy, suggesting strict monitoring for parameters that can be highly associated with clinical deterioration and adverse outcomes [38]. In addition to saturation < 95%, we also found levels of LDH ≥ 237 U/L and lymphocytes< 1.2 × 10^3^/μL as adequate targets for oxygen therapy.

Hospitalized patients aged<70y had a higher risk of being admitted to ICU if one of the following cases applied: ESRD, LDH ≥ 334 U/L, MDW ≥ 25, D-dimer≥2.37 mg/L and Lymphocytes< 0.7 × 10^3^/μL. Other studies found heart disease, COPD and heart rate to be significantly associated with ICU, in addition to ESRD [39]. The dominant role found for the latter in our model can be explained by the organ damage noticed in many patients admitted to ICU, which is associated with a high prevalence of limited renal function [40]. A high value of LDH is an indicator of tissue/cell destruction that is frequently used to monitor tissue damage associated with a wide range of disorders, including liver and interstitial lung disease. Patients with severe pulmonary interstitial disease present an increased LDH as one of the most important prognostic markers of lung injury [41, 42]. Lymphopenia is a biological disorder in patients with COVID-19 frequently considered as predictor of severe infection and myocardial injury, ARDS, and mortality [43]. Lymphopenia is a common consequence of infection caused by cytokine-induced reaction [44]. Reduced CD4+ T-cell and CD8+ T-cell levels promote viral replication and predict worsening outcome [45]. T -cells appear lower and functionally exhausted, and patients with COVID-19 with T- cells≤800/μL may still require urgent intervention due to a higher risk of further deterioration of their condition [46].

Several characteristics at entry to the ED were predictive of mortality in or out of hospital, independently from age and gender, including ESRD, procalcitonin≥0.2 ng/mL, LDH ≥ 307 U/L, saturation < 96% and D-dimer≥1.04 mg/L. We did not find known any significant predictive factor associated with increased mortality, differently from other reports addressing male gender, symptoms < 10 days prior to hospital admission, diabetes, coronary heart disease, chronic liver disease [47], white cell count, temperature, respiratory rate, lymphocytes and platelets [48, 49]. The significant association found between procalcitonin and mortality could be attributed to bacterial co-infection rather than viral replication [50].

Regarding our second research question, we were able to identify accurate risk scores, based on significant coefficients extracted from predictive models. The risk scores showed an average performance ranging from moderate to very high. The performance was very high for hospitalisation, with AUC = 91% (89–94%) and PPV = 94% (90–96%), mortality prediction, with an AUC = 91% (87–94%) and a low PPV = 60% (53–66%), as well as for oxygen support, with AUC = 87% (82–92%) and PPV = 92% (86–95%). The performance was slightly lower for admission to ICU, with AUC = 81% (73–89%) and PPV = 54% (43–65%). Notably, the NPV was high for death (95%, 92–97%) and admission to ICU (91%, 83–95%), indicating that a value of the score below the threshold, particularly for ICU, may be effective in ruling out major complications and considering oxygen therapy as a viable solution.

The comparison between predictive models estimated in our study and other scientific reports may be challenging. Predictive models use different types of cohorts to investigate a variety of outcomes including confirmed diagnosis, disease severity, ICU and mortality. Studies are conducted in different hospital environments, using different laboratory standards and applying heterogeneous inclusion/exclusion criteria e.g. tuberculosis, influenza and bronchitis [9]. Variables included in predictive models are also heterogeneous, from vital signs to image features, contact with other infected individuals, lymphocyte count, liver enzyme and red distribution width [9]. In many cases, data of non-hospitalized patients are limited and do not include laboratory and imaging analyses [51], drawing conclusions only from a limited set of characteristics, with an AUC as high as 90% [52].

To better respond to our second research question, we evaluated the accuracy of our predictive models under real life conditions during the second wave in late 2020.

We found that the overall performance was fairly robust for oxygen support, hospitalisation and death. On the other hand, the results were less satisfactory for admission to ICU (ranging between 66 and 81%, which corresponds to a sensitivity drop between 51 and 82%). A possible explanation may be related to the heterogeneous characteristics of patients admitted during the second wave, which changed the prognosis and thus the predictive ability of models specified under different conditions.

In summary, we identified a set of key parameters that can be translated into risk scores to inform clinical practice. The advantage of this approach is that it can be directly applied to the next patient entering the ED, even with a pocket calculator. A substantial barrier for the continuous update and adaptation of this method is the limited interoperability of health databases in most European contexts, which makes the process of data acquisition particularly burdensome. Improving the digitalization and standardization of health information at hospitals across Europe will be paramount to strengthen our preparedness to future outbreaks and favour the adoption of research methods in clinical practice [53–55].

Finally, several limitations of our study are worth to be outlined, along with their consequences on the future use of the algorithms.

Firstly, this is a retrospective study carried out at a single hospital, enrolling a limited number of patients during the initial phase of the COVID-19 outbreak in Italy. Consequently, the results may not equally apply to other jurisdictions and/or institutions. However, our report provides a focused stratification of subjects entering the ED that can be informative for clinical practice on a global scale.

Secondly, among the outcomes identified, only death represents a clinically objective measure, while all others reflect decisions made by clinicians at the hospital. Therefore, we cannot infer on the validity of the same models under different settings and variable conditions. However, it is a specific feature of the approach to be able to identify factors orienting practices, so that pragmatic guidelines can be offered when they are not readily available. This feature implies a repeated application of the method to make it locally relevant.

Thirdly, we searched for predictive variables among measures readily available at presentation to the ED. As for all observational studies, we may have missed a subset of characteristics that could be potentially predictive either at entry or in the subsequent follow up in and out of hospital. Nevertheless, we kept our focus on measures that are used in normal practice, so that they can be realistically applied.

Fourthly, the statistical models adopted did not take into account transitions between states e.g. oxygen support followed by intensive care. Our choice was based on the need to facilitate interpretation, rather than enhancing the statistical properties of our methods.

Finally, the stability of the algorithms should be considered in the broader perspective of a continuously evolving virus, in which the continuous update of the vaccination status may blunt the clinical response. This consideration triggers important closing reflections.

The vaccination status of individuals in our samples could not be ascertained from the available data. However, vaccination in Italy ramped up only in February–March 2021, reaching over 90% for those 60+ in early 2022 (see ECDC data at: https://vaccinetracker.ecdc.europa.eu/public/extensions/COVID-19/vaccine-tracker.html#uptake-tab). Therefore, while obviously vaccination was not an issue in 2020, it is also fair to assume that all patients included in the “Wild/Alpha” sample in Dec 2020-Jan 2021 were not vaccinated, while the majority of those included in the “Delta/Omicron” sample could be considered vaccinated. On this ground, we can fairly assume that the vaccination status may have biased estimates obtained for the Omicron sample. On the other hand, the fact that this characteristic is not continuously available in routine hospital databases makes it a difficult candidate for risk evaluation in everyday practice (other than asking directly to the patient, which may be prone to information bias). Therefore, in practical usage, it could be appropriate to consider it as a non- observable confounder, thus incorporating its effect in the association found for other variables.

Overall, the predictive models appeared moderately accurate, irrespective of the variant dominating the reference period. The AUC did not fall below 70%, except for intensive care, for which no variable was significant under the “Delta/Omicron” variants, and only ESRD was significant under the “Wild/Alpha” variants. This slight deviation may show that the subgroup of those below 70 years may not reflect practices following the initial emergency of the “Wild” outbreak (which is partially true also for oxygen support, where only lymphocytes retain their significance). Therefore, the method seems still generally useful to pick subjects at significantly increased risk.

The significance of specific variables in predictive models may also highlight differences in their relevance according to the lineage of the virus. The measurement of MDW, D Dimer, and Prothrombin did not appear as relevant in the evolution of COVID-19 for matters related to hospitalisation. On the other hand, saturation retained its predictive value for hospitalisation and oxygen therapy, while it was not significant to predict survival in the “Delta/Omicron” sample. These aspects seem to indicate changes in the characteristics of the population affected by different variants, reflecting the higher incidence of oxygen therapy and intensive care in the Wild/Alpha, as opposed to a sharp decrease in the incidence of all events under the Delta/Omicron variants (nearly 50% hospitalisations and deaths compared to other variants). On the other hand, the Wild/Alpha sample showed an increased 50% admission to intensive therapy, as opposed to the baseline population. Only few variables could be considered relevant for the new variants of COVID-19, primarily for hospitalisation (dyspnea, procalcitonin, saturation, reactive C protein) and death (ESRD, procalcitonin, LDH, D Dimer).

Since the model was estimated on the original virus, we may assume that our scoring algorithms will continue to be particularly relevant for any future new SARS-type respiratory diseases, where severe cases of pneumonia are more frequent in the absence of vaccination. The same model could be possibly continuously updated, estimating the weights of significant variables repeatedly over time.

## Conclusion

We identified demographic, clinical and laboratory parameters associated to hospitalisation, oxygen support, ICU admission and death in a population admitted to a large regional hospital with a confirmed diagnosis of COVID-19. Risk scores derived from multivariate models showed moderate to high predictive accuracy in flagging subjects with more severe prognosis, based upon the early evaluation of personal characteristics at the ED. The method can be conveniently applied to support clinical decisions under different conditions, using targeted data collection at a single point of entry. Recalibration of the scoring algorithms will be needed to cope with the continuous evolution of the virus in different contexts.

## Supplementary Information

**Additional file 1: Supplementary Table 1.** Validation of the Algorithms for the calculation of scores.

## Data Availability

The datasets analysed during the current study are available from the corresponding author on reasonable request. The above mentioned Pescara General Hospital Health Board has not provided explicit permission to publicise the release of individual patient records as open data.
