# Supplementary material for: Reliability of predictive models to support early decision making in the emergency department for patients with confirmed diagnosis of COVID-19: the Pescara Covid Hospital score
Source: BMC Health Serv Res. 2022 Aug 19;22:1062. doi: 10.1186/s12913-022-08421-4 (PMC9390116; doi:10.1186/s12913-022-08421-4)
Supplement: Supplementary file 1 — Additional file 1: Supplementary Table 1. Validation of the Algorithms for the calculation of scores. [file 12913_2022_8421_MOESM1_ESM.doc]

**Supplementary Table 1. Validation of the Algorithms for the calculation of scores**

|  | **Hospitalisation** | **Oxygen Support <70 yrs** | **Intensive Therapy <70 yrs** | **Death** |
| --- | --- | --- | --- | --- |
| **Algorithm**  **Condition value:**  **[1=Yes; 0=No]** | round( 0.02 * [age]) +  + 1 * [Male]  + 3 * [Diabetes]  + 2 * [Dyspnea]  + 3 * [Procalcitonin **≥**0.2 ng/mL]  + 4 * [MDW **≥** 22]  + 3 * [Saturation ≤ 95%]  + 2 * [D-dimer **≥** 0.72 mg/L]  + 2 * [Prothrombin time **<** 95%]  + 3 * [CRP **≥** 21 mg/L] | 6 * [LDH **≥**237 U/L]  + 11 * [Saturation < 95%]  + 4 * [Lymphocytes < 1.2x10^3^/µL] | round(0.05 * [Age]) +  + 59 * [ESRD]  + 6 * [LDH **≥** 334 U/L]  + 3 * [MDW **≥** 25]  + 5 * [D-dimer **≥** 2.37 mg/L]  + 3 * [Lymphocytes < 0.7 x10^3^/µL] | round(0.06* [Age]) +  + 2* [ESRD])  + 3* [Procalcitonin **≥** 0.2 ng/mL])  + 2* [LDH **≥** 307 U/L])  + 3* [Saturation < 96%])  + 2* [D-dimer **≥**1.04 mg/L]) |
| **Optimal Cutpoint** | **≥12** | **≥8** | **≥10** | **≥10** |
| **External validation (“Delta”: December 2020 - January 2021)** | | | | |
| **Confusion Matrix** | |  | Yes | No |  | | --- | --- | --- | --- | | Score + | 126 | 14 | 140 | | Score - | 26 | 50 | 76 | | Total | 152 | 64 | 216 | | |  | Yes | No |  | | --- | --- | --- | --- | | Score + | 76 | 1 | 77 | | Score - | 10 | 4 | 14 | | Total | 86 | 5 | 91 | | |  | Yes | No |  | | --- | --- | --- | --- | | Score + | 22 | 10 | 32 | | Score - | 21 | 41 | 62 | | Total | 43 | 51 | 94 | | |  | Yes | No |  | | --- | --- | --- | --- | | Score + | 36 | 46 | 82 | | Score - | 15 | 120 | 135 | | Total | 51 | 166 | 217 | |
| **Youden Index** | 0.61 | 0.68 | 0.32 | 0.43 |
| **Sensitivity** | **0.83 ( 0.76 - 0.88 )** | **0.88 ( 0.80 - 0.94 )** | **0.51 (0.37-0.65)** | **0.71 ( 0.57 - 0.81 )** |
| **Specificity** | **0.78 ( 0.67 - 0.86 )** | **0.80 ( 0.38 - 0.96 )** | **0.80 (0.67-0.89)** | **0.72 ( 0.65 - 0.78 )** |
| **PPV** | 0.90 ( 0.84 - 0.94 ) | 0.99 ( 0.93 - 1.00 ) | 0.69 (0.51-0.82) | 0.44 ( 0.34 - 0.55 ) |
| **NPV** | 0.66 ( 0.55 - 0.75 ) | 0.29 ( 0.12 - 0.55 ) | 0.66 (0.54-0.77) | 0.89 ( 0.82 - 0.93 ) |
| **LR+** | 3.79 ( 2.45 – 6.69 ) | 4.42 ( 1.44 - Inf ) | 2.61 (1.44-5.61) | 2.55 ( 1.88 - 3.50 ) |
| **LR-** | 0.22 ( 0.14 - 0.31 ) | 0.14 (0.09 - 0.37 ) | 0.61 (0.41-0.82) | 0.41 ( 0.24 - 0.60 ) |
| **Accuracy** | 0.81 | 0.88 | 0.66 | 0.72 |
| **AUC** | **0.81 ( 0.75 – 0.87 )** | **0.84 ( 0.71 – 0.97 )** | **0.66 ( 0.55 – 0.77 )** | **0.71 ( 0.63 – 0.80 )** |
| **External validation (“Omicron”: January-March 2022)** | | | | |
| **Confusion Matrix** | |  | Yes | No |  | | --- | --- | --- | --- | | Score + | 97 | 62 | 155 | | Score - | 15 | 125 | 144 | | Total | 112 | 187 | 299 | | |  | Yes | No |  | | --- | --- | --- | --- | | Score + | 28 | 4 | 32 | | Score - | 7 | 7 | 14 | | Total | 35 | 11 | 46 | | |  | Yes | No |  | | --- | --- | --- | --- | | Score + | 5 | 14 | 19 | | Score - | 2 | 20 | 22 | | Total | 7 | 34 | 41 | | |  | Yes | No |  | | --- | --- | --- | --- | | Score + | 33 | 77 | 110 | | Score - | 2 | 149 | 151 | | Total | 35 | 226 | 261 | |
| **Youden Index** | 0.54 | 0.43 | 0.30 | 0.60 |
| **Sensitivity** | **0.87 ( 0.79 - 0.92 )** | **0.80 ( 0.64 - 0.90 )** | **0.71 ( 0.36-0.92 )** | **0.94 ( 0.81 - 0.98 )** |
| **Specificity** | **0.67 ( 0.60 - 0.73 )** | **0.63 ( 0.35 - 0.85 )** | **0.59 ( 0.42-0.74 )** | **0.66 ( 0.60 - 0.72 )** |
| **PPV** | 0.61 ( 0.53 - 0.68 ) | 0.87 ( 0.72 – 0.95 ) | 0.26 ( 0.12 - 0.49 ) | 0.30 ( 0.22 - 0.39 ) |
| **NPV** | 0.89 ( 0.83 - 0.93 ) | 0.50 ( 0.27 - 0.73 ) | 0.91 ( 0.72 - 0.97 ) | 0.99 ( 0.95 - 0.99 ) |
| **LR+** | 2.61 ( 2.13 – 3.29 ) | 2.20 ( 1.19 - 9.07 ) | 1.73 ( 0.74 – 3.08 ) | 2.77 ( 2.73 – 3.40 ) |
| **LR-** | 0.20 ( 0.11 - 0.31 ) | 0.31 ( 0.20 - 0.55 ) | 0.49 ( 0.00 – 1.17 ) | 0.09 ( 0.00 - 0.23 ) |
| **Accuracy** | 0.74 | 0.83 | 0.61 | 0.70 |
| **AUC** | **0.77 ( 0.71 – 0.83 )** | **0.72 ( 0.56 – 0.88 )** | **0.65 ( 0.41 - 0.89)** | **0.80 ( 0.71 - 0.89)** |
